# Brassinolide Application Mitigates Blossom-End Rot in Tomato by Enhancing Calcium Homeostasis and Antioxidant Defense Under Calcium Deficiency

**DOI:** 10.3390/plants15030427

**Published:** 2026-01-30

**Authors:** Dandan Wang, Xingqiang Fan, Lingdi Dong, Yan Li, Yikang Xue, Hongyu Li, Qingyin Zhang, Lianfen Qi, Yansu Li

**Affiliations:** 1 Shijiazhuang Academy of Agriculture and Forestry Sciences, Shijiazhuang 050041, China; yuwenhanzhu@126.com (D.W.); yoginlily@126.com (Y.L.); 15502412520@163.com (Y.X.); nkylhyy@163.com (H.L.); xli760103@163.com (Q.Z.); 2 Institute of Vegetables and Flowers, Chinese Academy of Agricultural Sciences, Beijing 100081, China; fanxingqiang@caas.cn; 3 Institute of Cash Corps, Hebei Academy of Agriculture and Forestry Sciences, Shijiazhuang 050051, China; donglingdi@163.com

**Keywords:** tomato, BER, BR, calcium absorption, antioxidant system, transcriptome analysis

## Abstract

Blossom-end rot (BER) in tomatoes is a physiological disorder primarily caused by the disruption of calcium absorption and transport. This study cultivated tomatoes using a trough-based vermiculite system. Two treatments were established: a calcium-deficient nutrient solution and a calcium-deficient nutrient solution supplemented with 0.1 mg/L BR (*n* = 40 plants per treatment). The activities of catalase (CAT), peroxidase (POD), and superoxide dismutase (SOD), as well as the contents of malondialdehyde (MDA) and hydrogen peroxide (H_2_O_2_), were measured in the leaves. Calcium ion content was also determined in various plant parts. Statistical analysis of differences was performed using Duncan’s multiple range test at a significance level of *p* < 0.01. Concurrently, transcriptome sequencing of root, stem, and leaf tissues was conducted via high-throughput sequencing technology. The results showed that foliar application of BR under calcium deficiency significantly reduced the incidence of BER (from 26.67% to 6.67%) and effectively increased calcium ion content in leaves, stems, and roots. At the physiological level, BR treatment markedly enhanced the activities of CAT, POD, and SOD in leaves (by 105.70%, 117.12%, and 82.77%, respectively), while reducing H_2_O_2_ and MDA contents (by 36.90% and 16.38%, respectively). This indicates that BR alleviates membrane lipid peroxidation damage by enhancing the antioxidant defense system. Gene Ontology (GO) enrichment analysis revealed that the differentially expressed genes (DEGs) were primarily involved in biological processes, such as secondary metabolic processes, response to oxygen-containing compounds, and cell wall organization. KEGG pathway analysis further indicated significant enrichment in pathways, including phenylpropanoid biosynthesis, plant hormone signal transduction, and plant–pathogen interaction. Additionally, key genes, such as the cytochrome c oxidase (COX) gene (Solyc03g013460.1), exhibited a gradient up-regulation pattern (root > stem > leaf) in the oxidative phosphorylation pathway. In conclusion, BR likely enhances tomato tolerance to calcium deficiency stress and effectively reduces BER incidence through multiple pathways: regulating calcium absorption and distribution, activating the antioxidant system, modulating hormone signaling pathways, and enhancing energy metabolism. These findings provide a theoretical basis for the application of BR in agricultural production.

## 1. Introduction

As a high-yielding crop, tomato (*Solanum lycopersicum*) holds significant global economic value [1]. It is also widely recognized as one of the most important vegetable crops worldwide [2]. In production, BER is a common physiological disorder that causes black spots or even rot on fruits, severely constraining the improvement of tomato yield and quality. This physiological disorder is not unique to tomatoes; it also occurs in crops such as peppers, eggplants, watermelons, and chestnut [3,4,5,6].

Low soil calcium content is one factor associated with increased BER incidence [7]. However, increasing plant calcium uptake by augmenting soil calcium does not necessarily lead to increased fruit calcium uptake or reduced BER [8]. Calcium is an essential mineral element crucial for plant growth and development, playing key roles in stabilizing cell wall structure, maintaining membrane system integrity, regulating enzyme activity, and acting as an intracellular second messenger [9]. The transport and spatial distribution of calcium within plants are unique, primarily following the “xylem pathway.” This process is strongly dependent on transpiration and directly leads to significant accumulation of calcium in organs with high transpiration rates, such as leaves [10]. Once calcium ions absorbed by the roots enter the xylem, their upward transport relies almost entirely on the driving force of transpirational pull. Calcium ions are passively transported with the flow of xylem sap, moving from the roots through the stems to various aboveground organs [11]. Differences in transpiration intensity among organs directly determine their ability to acquire calcium via the xylem [10]; leaves, as the primary transpirational organs, exhibit sustained and vigorous transpirational flow. Consequently, a substantial amount of calcium delivered by the xylem is unloaded and accumulates here to meet their physiological needs. In contrast, fruit surfaces are often covered with cuticles or wax layers, have few stomata, and exhibit very low transpiration rates. Thus, even when root uptake and overall xylem transport of calcium are sufficient, fruits struggle to acquire adequate calcium through the xylem stream. This spatial disconnection between supply and demand is the fundamental cause of physiological disorders such as BER in tomatoes [9]. Calcium is a nutrient with poor mobility. Orchard applications proved that both foliar spraying and root irrigation of Ca-encapsulated carbon dots could significantly enhance Ca content in apple fruit [12]. Foliar application of calcium can reduce BER in tomatoes [13], possibly due to direct calcium absorption into the fruit. Applying appropriate amounts of calcium to tomato plants effectively reduces the incidence and severity of BER, but compared to appropriate application, excessive calcium does not reduce incidence and may even further increase severity [8]. Rapid fruit growth and cell expansion are another factor contributing to BER development [14,15]. Aslani et al. [16] found a significant correlation between BER incidence and tomato fruit growth rate, but no significant correlation between fruit calcium content and BER incidence.

Plant hormones are key regulatory factors. Although present in minute amounts, they significantly influence the entire plant life cycle, including seed germination and the development of vegetative and reproductive organs. Furthermore, they play roles in responding to abiotic stresses, such as drought, salinity, and low temperature, helping plants enhance stress resistance and maintain normal physiological activities. Studies have found that exogenously applied plant growth regulators can also regulate the occurrence and development of BER. Auxin and gibberellin (GA), while accelerating fruit growth, also increase BER incidence [17]. Foliar spraying of 500 mg·L^−1^ abscisic acid (ABA) significantly reduces BER occurrence [18]. BR, as an important plant hormone, has recently attracted widespread attention in the scientific community for its role in regulating plant growth and development, especially in application research on vegetable crops like tomatoes. BR demonstrates significant physiological effects, including promoting plant growth, enhancing stress resistance, and improving fruit quality [19,20]. Exogenous BR (0.5 μM) effectively reduces the incidence and disease index of internal tipburn in mini Chinese cabbage. It up-regulates the expression of genes related to calcium ion transfer, response, and binding, increases the activity of calcium ion transporter Ca^2+^-ATPase and proton pump H^+^-ATPase, and promotes calcium transport from underground to aboveground parts and from outer to inner leaves, thereby alleviating internal tipburn symptoms caused by calcium deficiency in inner leaves [21]. BR can reduce tomato sensitivity to BER. After EBL treatment, soluble Ca^2+^ and the activities of three major antioxidant enzymes (ascorbate peroxidase, catalase, and superoxide dismutase) in tomato fruits significantly increased, thereby reducing BER occurrence [22].

In our preliminary studies, we observed that BR-treated tomatoes exhibited less incidence of BER compared to those not treated with BR. This result suggests that BR may be involved in tomato resistance to BER. The aim of this study is to investigate the role of BR and its potential underlying mechanisms in tomato’s defense against BER.

## 2. Results

### 2.1. Plant Growth Status Under Calcium-Deficient Nutrient Solution + Distilled Water Spray and Calcium-Deficient Nutrient Solution + BR Spray

**Figure 1:**
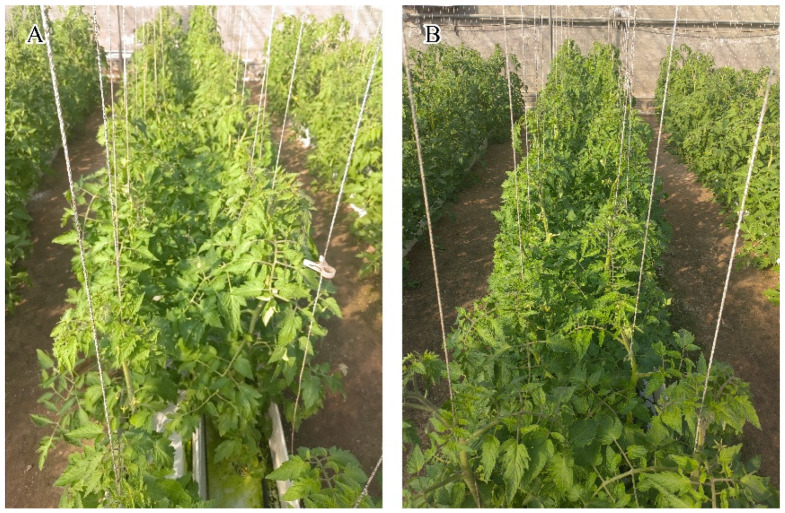
Tomato plant growth status. (**A**) Calcium-deficient nutrient solution cultivation + distilled water spray treatment. (**B**) Calcium-deficient nutrient solution cultivation + BR spray treatment.

**Figure 2:**
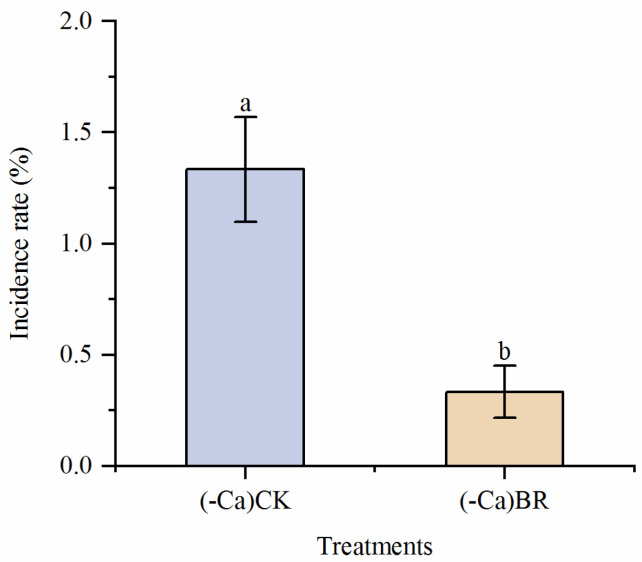
Initial BER incidence in tomatoes under calcium-deficient nutrient solution + distilled water spray and calcium-deficient nutrient solution + BR spray treatments. Different lowercase letters indicated statistical differences (*p* < 0.01) as evaluated by Duncan’s multiple range test method.

**Figure 3:**
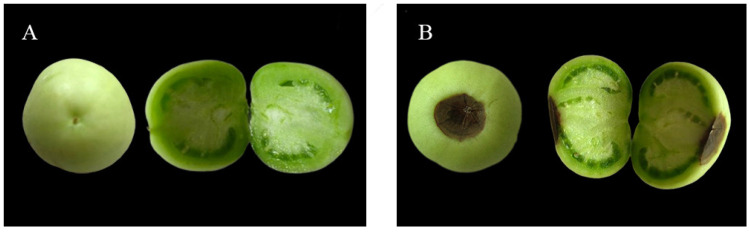
Tomato fruit growth status. (**A**) Top view and longitudinal section of a normal fruit. (**B**) Top view and longitudinal section of a BER-affected fruit.

At sampling time, the plant growth status of (-Ca) CK and (-Ca) BR treatments was similar, with no significant difference, as shown in Figure 1. The fruit growth status differed markedly between (-Ca) CK and (-Ca) BR treatments. The initial BER incidence in (-Ca) CK tomatoes was 26.67%, while in (-Ca) BR it was 6.67%, indicating that BR spraying under calcium deficiency significantly reduced BER occurrence (Figure 2). Figure 3 shows top and longitudinal section views of BER-affected and normal tomato fruits.

### 2.2. Effect of BR on Calcium Ion Content in Different Tomato Parts

**Figure 4:**
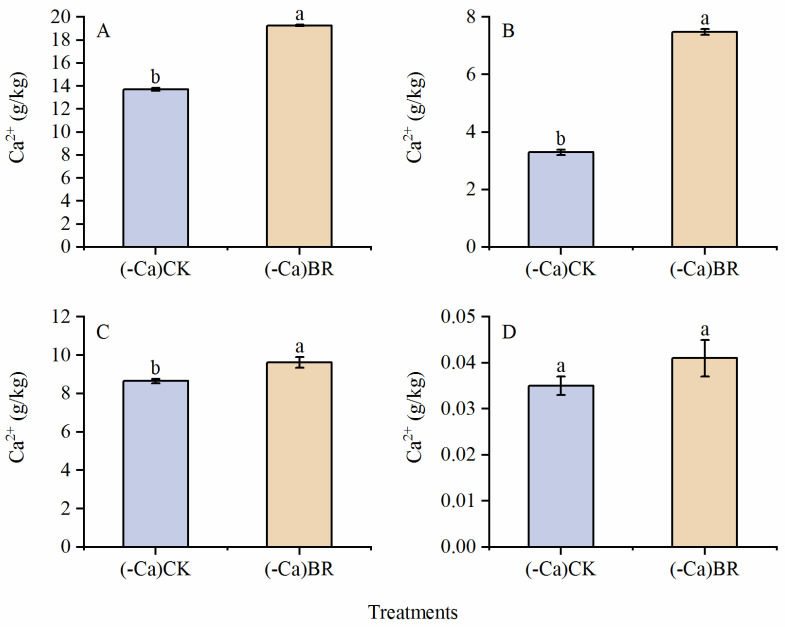
Calcium ion content in different tomato parts: (**A**) in leaves; (**B**) in stems; (**C**) in roots; and (**D**) in fruits. Different lowercase letters indicated statistical differences (*p* < 0.01) as evaluated by Duncan’s multiple range test method.

Compared to the control, BR spraying significantly increased Ca^2+^ content in the leaves, stems, and roots of tomato plants, although the increase observed in fruits was not statistically significant. Specifically, the BR treatment led to increases of 40.39%, 127.14%, 11.27%, and 17.14% in the leaves, stems, roots, and fruits, respectively (Figure 4).

### 2.3. Effect of BR on Antioxidant Enzymes in Tomato Leaves

**Figure 5:**
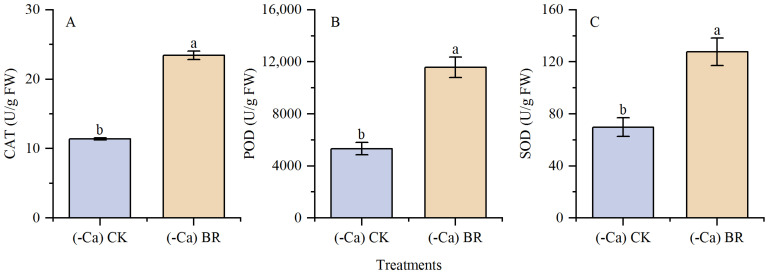
Effect of BR on the antioxidant enzyme system in tomato leaves. (**A**) CAT enzyme activity. (**B**) POD enzyme activity. (**C**) SOD enzyme activity. Different lowercase letters indicated statistical differences (*p* < 0.01) as evaluated by Duncan’s multiple range test method.

CAT, POD, and SOD are the most important enzymes in the antioxidant system. During cultivation with a calcium-deficient nutrient solution, exogenous BR spraying statistically significantly increased the activities of CAT, POD, and SOD by 105.70%, 117.12%, and 82.77%, respectively, compared to foliar spraying of distilled water (Figure 5). This indicates that exogenous BR spraying can effectively reduce membrane lipid peroxidation and protect cell membrane integrity.

### 2.4. Effect of BR on MDA in Tomato Leaves

**Figure 6:**
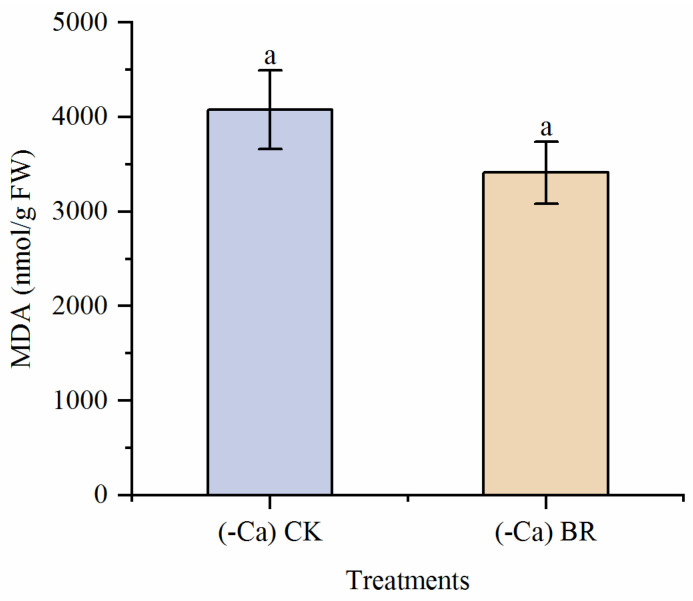
Effect of BR on MDA content in tomato leaves. Different lowercase letters indicated statistical differences (*p* < 0.01) as evaluated by Duncan’s multiple range test method.

Under calcium-deficient conditions, MDA content accumulated in leaves. Foliar spraying of BR reduced MDA content by 16.38%, but this reduction was not statistically significant (Figure 6). This indicates that exogenous BR has a certain regulatory effect on membrane damage in leaves.

### 2.5. Effect of BR on H_2_O_2_ in Tomato Leaves

**Figure 7:**
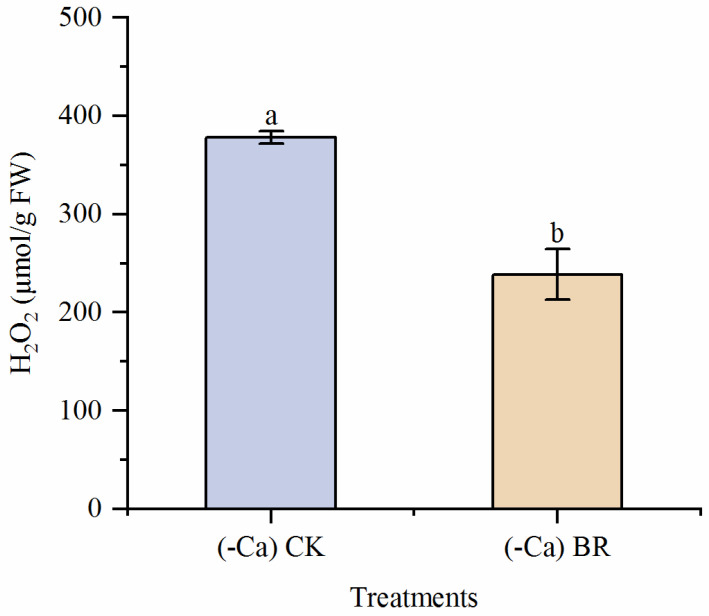
Effect of BR on H_2_O_2_ content in tomato leaves. Different lowercase letters indicated statistical differences (*p* < 0.01) as evaluated by Duncan’s multiple range test method.

Under calcium-deficient conditions, H_2_O_2_ content accumulated significantly in leaves. Exogenous BR spraying statistically significantly reduced H_2_O_2_ content in tomato leaves by 36.90% (Figure 7), indicating that exogenous BR reduces toxicity in BER-affected tomatoes by decreasing oxidative damage.

### 2.6. Differential Gene Screening

**Figure 8:**
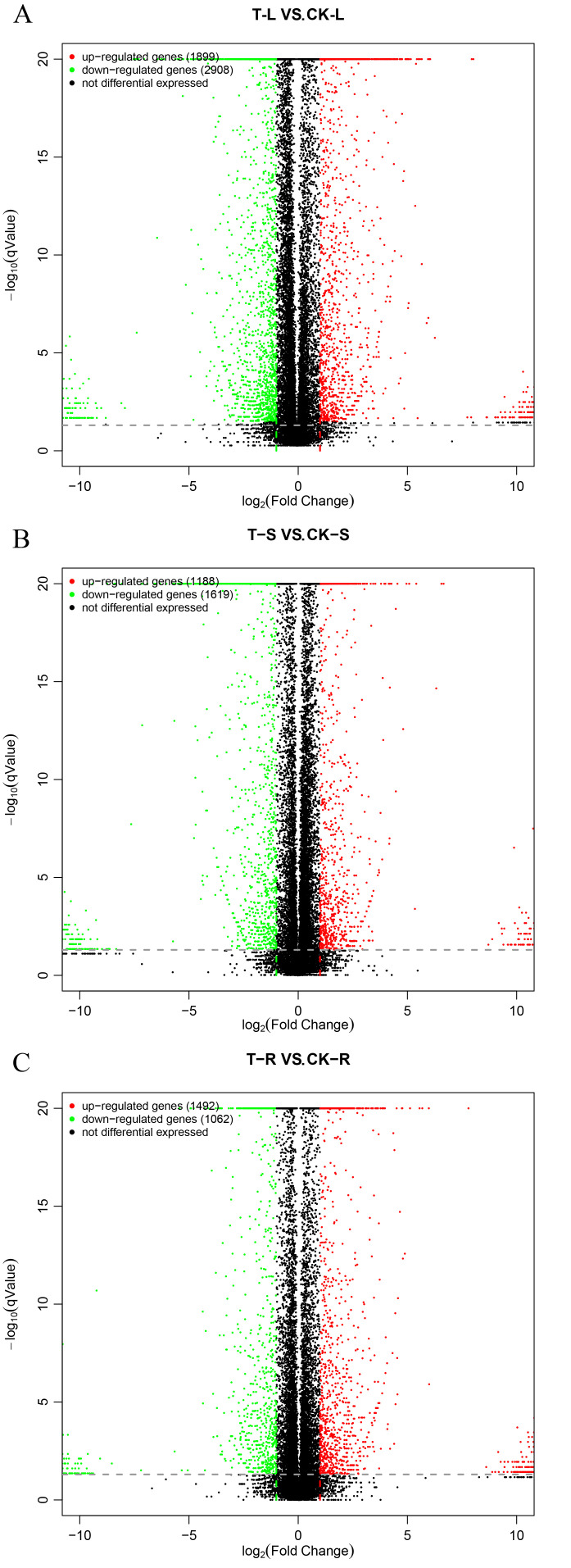
Volcano plots of differentially expressed genes. (**A**) DEGs in leaves. (**B**) DEGs in stems. (**C**) DEGs in roots. The DEGs were obtained by *p*-value ≤ 0.05, q-value ≤ 0.05, and |log_2_ Fold Change| ≥ 1.

Transcriptome sequencing data were used to compare samples from different tomato parts. The PCA plot is shown in Supplementary Figure S1. DEGs were screened with *p*-value ≤ 0.05, q-value ≤ 0.05, and |log_2_FoldChange| ≥ 1. The volcano plot results are shown in Figure 8. T-L_vs_CK-LTranscriptome analysis identified differential gene expression across all tissues. In leaves (T-L vs. CK-L), 4807 DEGs were identified (1899 up- and 2908 down-regulated). Similarly, 2807 DEGs were found in stems (T-S vs. CK-S; 1188 up, 1619 down), and 2554 in roots (T-R vs. CK-R; 1492 up, 1062 down). This suggests that more genes are involved, and more complex biological changes occur during leaf development, followed by stems, with the fewest DEGs in roots.

To further elucidate the mechanism by which BR controls tomato BER, high-throughput sequencing was used to study the expression patterns of functional genes in leaves, stems, and roots after BR treatment. Preliminary transcriptome results showed that BR treatment induced differential gene expression across all tissues. The comparison T-L vs. CK-L revealed 472 up-regulated and 517 down-regulated DEGs (|log_2_FC| ≥ 3) (Table 1). In stems (T-S vs. CK-S), 238 genes were up-regulated and 433 were down-regulated (Table 2). Similarly, in roots (T-R vs. CK-R), we identified 466 up-regulated and 294 down-regulated DEGs (Table 3).

### 2.7. GO Enrichment Analysis of Differentially Expressed Genes

**Figure 9:**
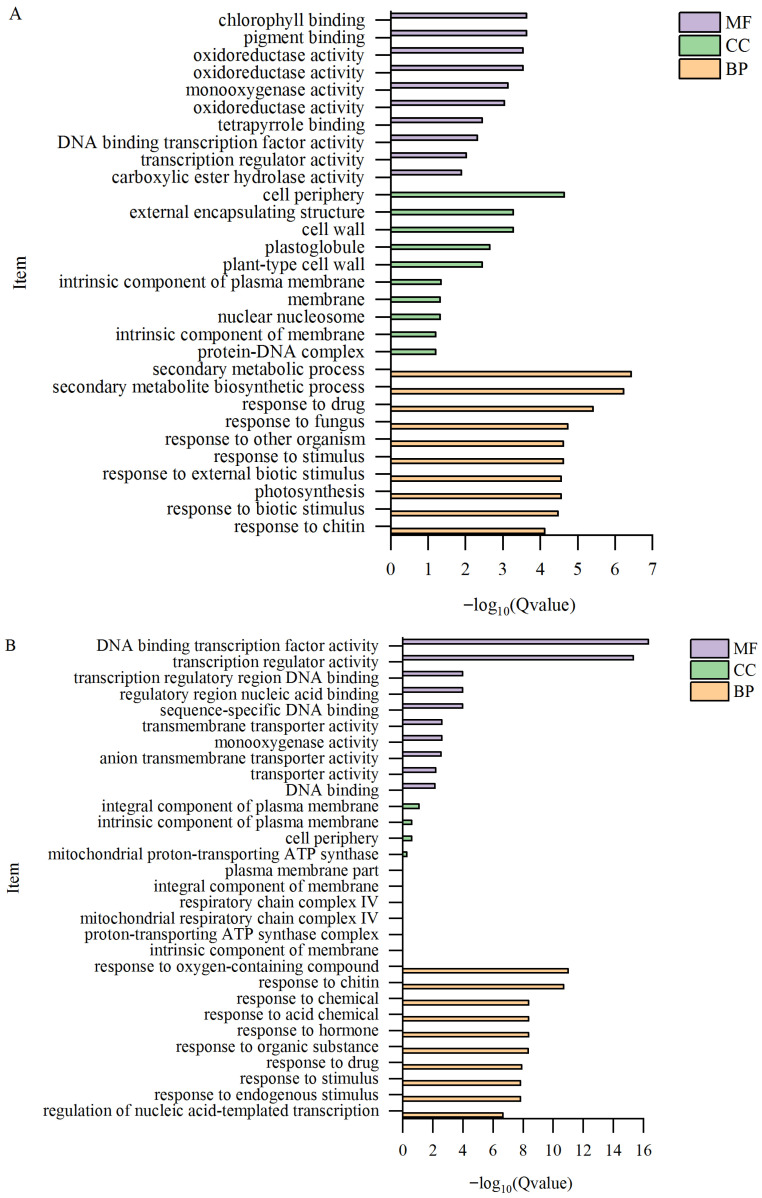

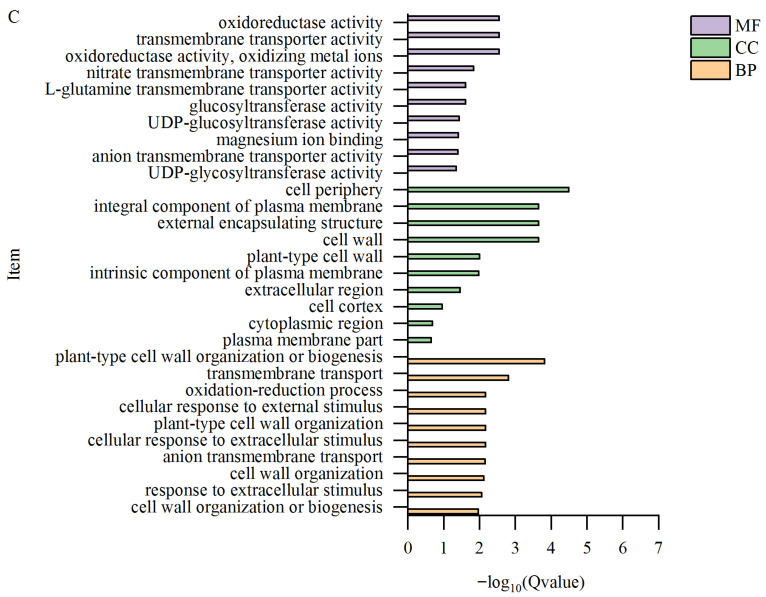
GO functional annotation results of DEGs in different comparison groups. (**A**) Leaf. (**B**) Stem. (**C**) Root.

GO functional annotation results of DEGs for biological process (BP), cellular component (CC), and molecular function (MF) are shown. Among the three comparison groups, the BP category contained the most enriched DEGs (Table 4). The top 10 GO terms with the smallest Q-value and most significant enrichment were selected for plotting (Figure 9). The most significantly enriched BP terms differed by tissue: secondary metabolic process for T-L vs. CK-L (Figure 9A), response to oxygen-containing compounds for T-S vs. CK-S (Figure 9B), and plant-type cell wall organization or biogenesis for T-R vs. CK-R (Figure 9C). The most significantly enriched pathways in CC were extracellular region, integral component of plasma membrane, and extracellular region. The most significantly enriched pathways in MF were chlorophyll binding, DNA-binding transcription factor activity, and oxidoreductase activity.

### 2.8. KEGG Metabolic Pathway Enrichment Analysis of Differentially Expressed Genes

KEGG functional enrichment was performed on DEGs, and the top 30 significantly enriched metabolic pathways were screened. Phenylpropanoid biosynthesis, plant hormone signal transduction, and plant-pathogen interaction were the most abundant pathways across the three comparison groups.

**Figure 10:**
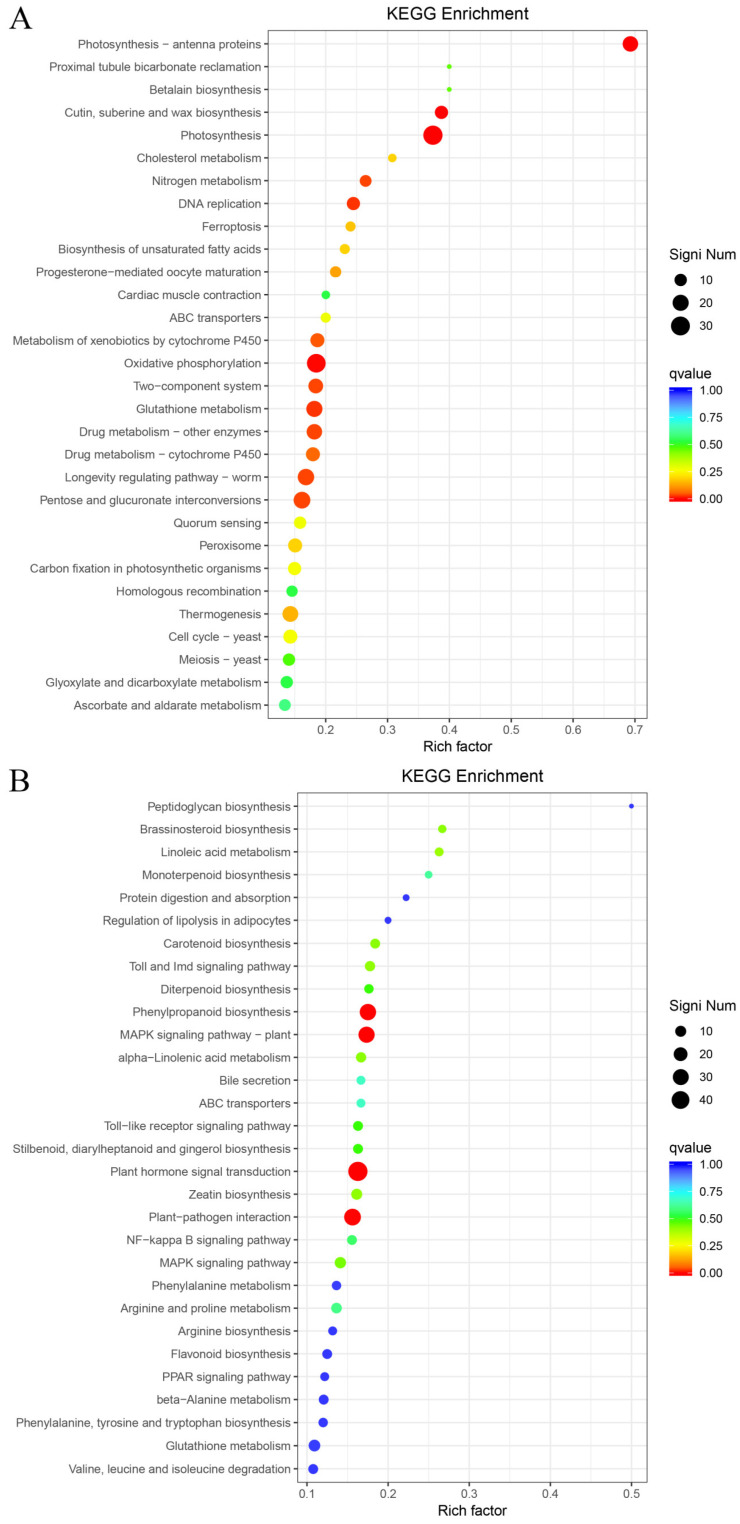
KEGG enrichment analysis plots for T-L vs. CK-L. (**A**) Up-regulated. (**B**) Down-regulated.

Among the significantly up-regulated metabolic pathways in the T-L vs. CK-L comparison, the most significantly enriched were photosynthesis-antenna proteins (ko00196, 18 genes), cutin, suberine and wax biosynthesis (ko00073, 12 genes), photosynthesis (ko00195, 31 genes), and oxidative phosphorylation (ko00190, 29 genes) (Figure 10A). Among the significantly down-regulated metabolic pathways, the most significantly enriched were phenylpropanoid biosynthesis (ko00940, 32 genes), MAPK signaling pathway-plant (ko04016, 31 genes), plant hormone signal transduction (ko04075, 49 genes), and plant-pathogen interaction (ko04626, 34 genes) (Figure 10B).

**Figure 11:**
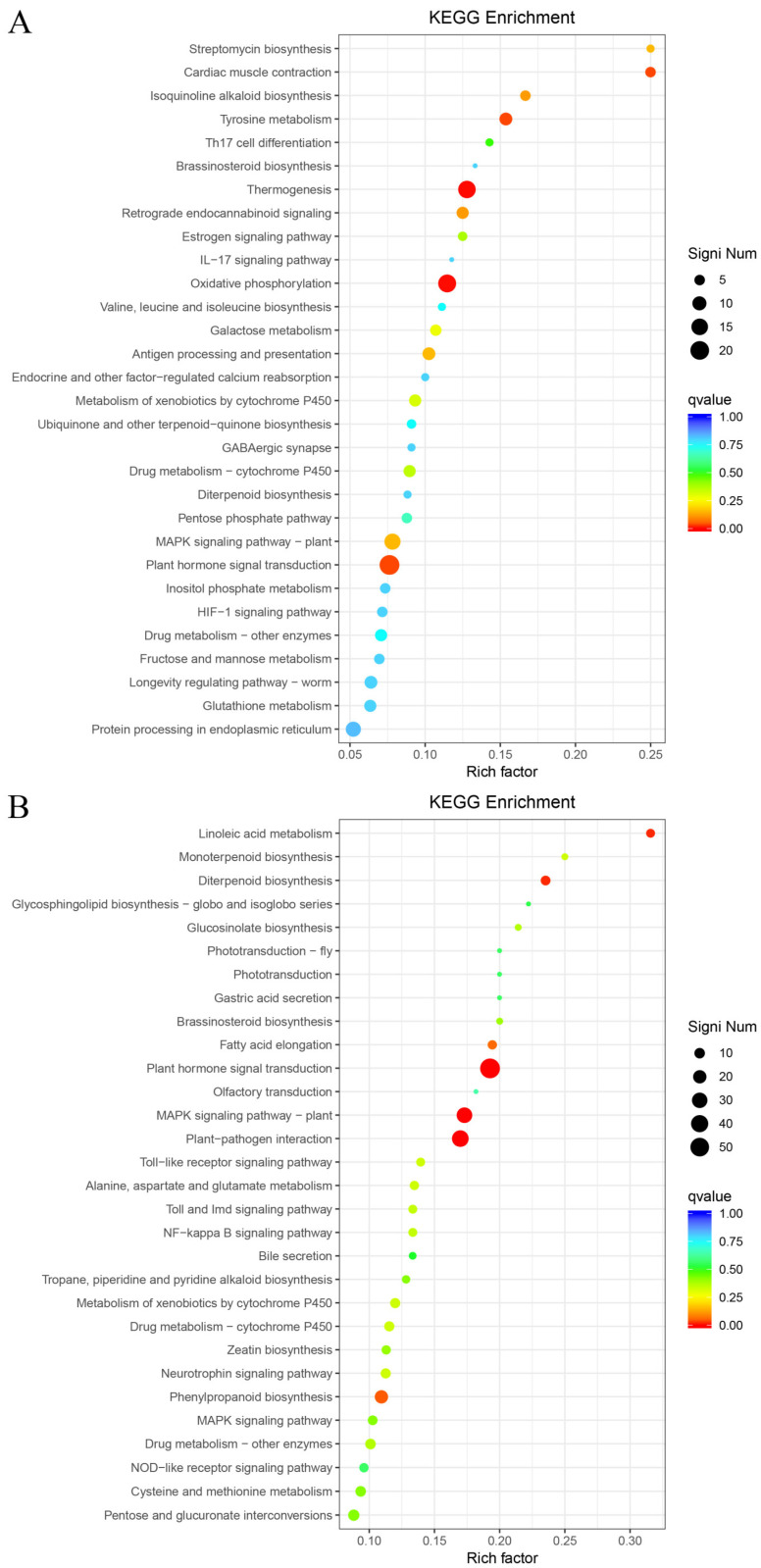
KEGG enrichment of DEGs in T-S vs. CK-S. (**A**) Up-regulated. (**B**) Down-regulated.

Among the significantly up-regulated metabolic pathways in the T-S vs. CK-S comparison, the most significantly enriched were thermogenesis (ko04714, 17 genes) and oxidative phosphorylation (ko00190, 18 genes) (Figure 11A). Among the significantly down-regulated metabolic pathways, the most significantly enriched were linoleic acid metabolism (ko00591, 6 genes), diterpenoid biosynthesis (ko00904, 8 genes), plant hormone signal transduction (ko04075, 58 genes), MAPK signaling pathway-plant (ko04016, 31 genes), and plant-pathogen interaction (ko04626, 37 genes) (Figure 11B).

**Figure 12:**
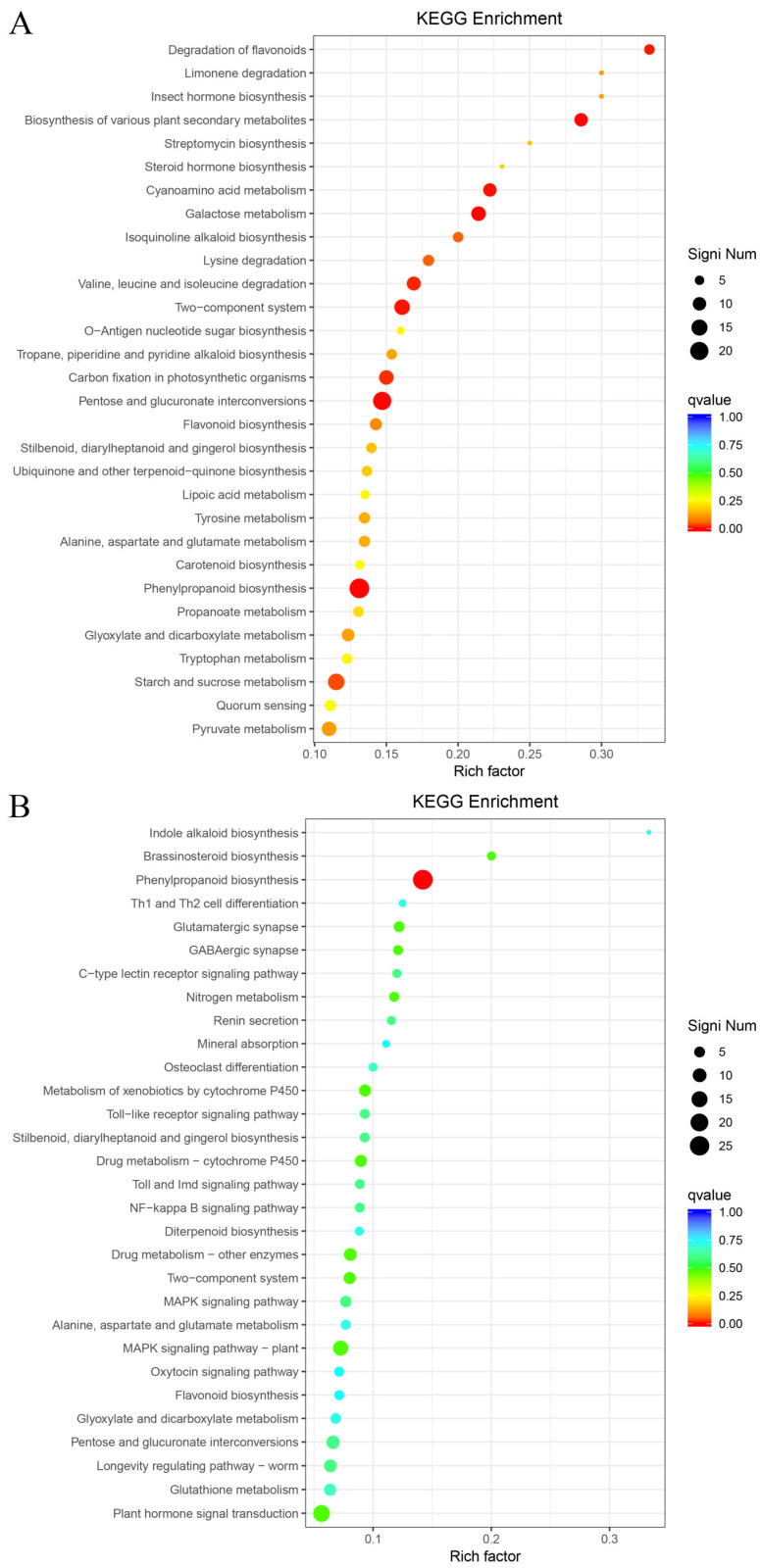
KEGG enrichment of DEGs in T-R vs. CK-R. (**A**) Up-regulated. (**B**) Down-regulated.

Among the significantly up-regulated metabolic pathways in the T-R vs. CK-R comparison, the most significantly enriched were degradation of flavonoids (ko00946, 6 genes), biosynthesis of various plant secondary metabolites (ko00999, 10 genes), cyanoamino acid metabolism (ko00460, 10 genes), galactose metabolism (ko00052, 12 genes), valine, leucine and isoleucine degradation (ko00280, 11 genes), two-component system (ko02020, 14 genes), pentose and glucuronate interconversions (ko00040, 20 genes), and phenylpropanoid biosynthesis (ko00940, 24 genes) (Figure 12A). Among the significantly down-regulated metabolic pathways, the most significantly enriched was phenylpropanoid biosynthesis (ko00940, 26 genes) (Figure 12B).

### 2.9. Metabolic Pathways

**Figure 13:**
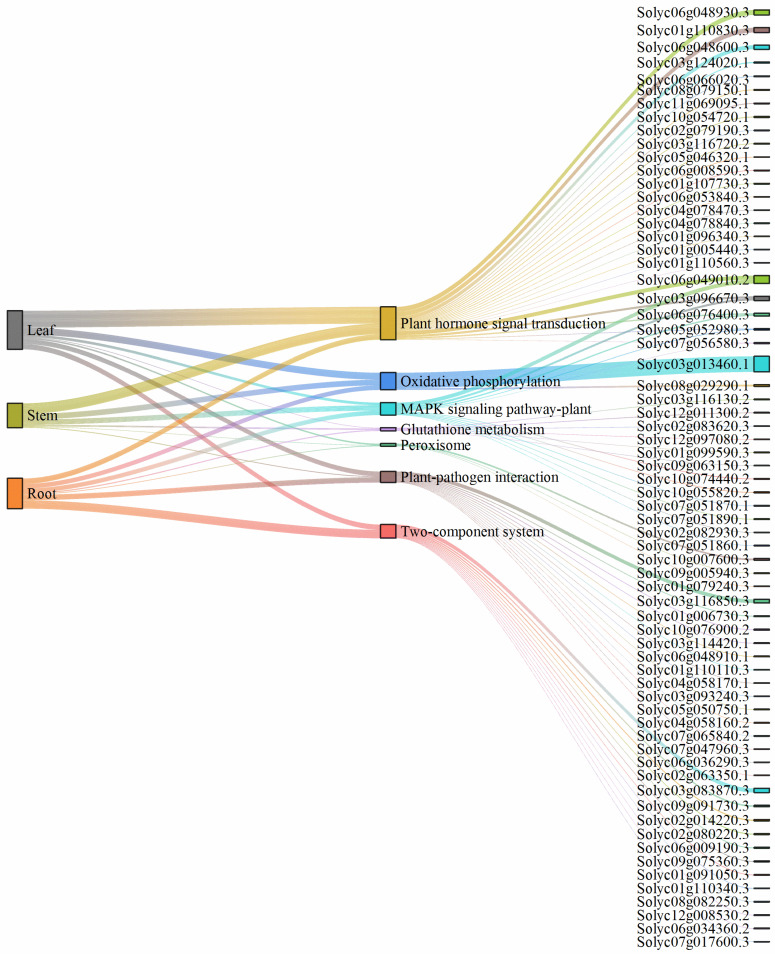
Sankey diagram of related up-regulated expressed genes in tomato roots, stems, and leaves.

Figure 13 shows up-regulated differentially expressed genes related to the occurrence of tomato BER in roots, stems, and leaves, involving pathways such as plant hormone signal transduction, oxidative phosphorylation, MAPK signaling pathway-plant, glutathione metabolism, peroxisome, plant-pathogen interaction, and two-component system. In the hormone signal transduction pathway, genes related to jasmonic acid, auxin-responsive protein SAUR, and protein phosphatase PP2C were more highly up-regulated in leaves, stems, and roots. Among these, Solyc06g048600.3 and Solyc06g048930.3 showed the highest up-regulation in leaves, with |log_2_ Fold Change| values of 10.87 and 13.41, respectively. Solyc01g110830.3 showed the highest up-regulation in stems (log_2_ Fold Change = 12.78), and Solyc06g049010.2 showed the highest up-regulation in roots (log_2_ Fold Change = 10.66). In the oxidative phosphorylation metabolic pathway, COX showed significant up-regulation in leaf, stem, and root tissues. The gene encoding this enzyme, Solyc03g013460.1, exhibited a strong tissue-specific up-regulation trend, with |log_2_ Fold Change| values of 15.28, 13.57, and 12.18 in roots, stems, and leaves, respectively. In the MAPK signaling pathway–plant metabolic pathway, genes related to protein phosphatase PP2C and endochitinase were most highly up-regulated in roots, stems, and leaves, with Solyc06g049010.2 showing the highest up-regulation in roots (log_2_ Fold Change = 10.66). In the glutathione metabolism pathway, GST was most highly up-regulated in leaves, stems, and roots. In the peroxisome pathway, glycolate oxidase and fatty acyl-CoA were most highly up-regulated in leaves, stems, and roots. In the plant–pathogen interaction pathway, genes related to calcium-dependent protein, calmodulin, and calcium-binding protein were most highly up-regulated in leaves, stems, and roots, with Solyc03g116850.3 showing the highest up-regulation in roots (log_2_ Fold Change = 10.06). In the two-component system pathway, genes related to pectinesterase and endoglucanase were most highly up-regulated in leaves, stems, and roots, with Solyc03g083870.3 showing the highest up-regulation in roots (log_2_ Fold Change = 11.23).

**Figure 14:**
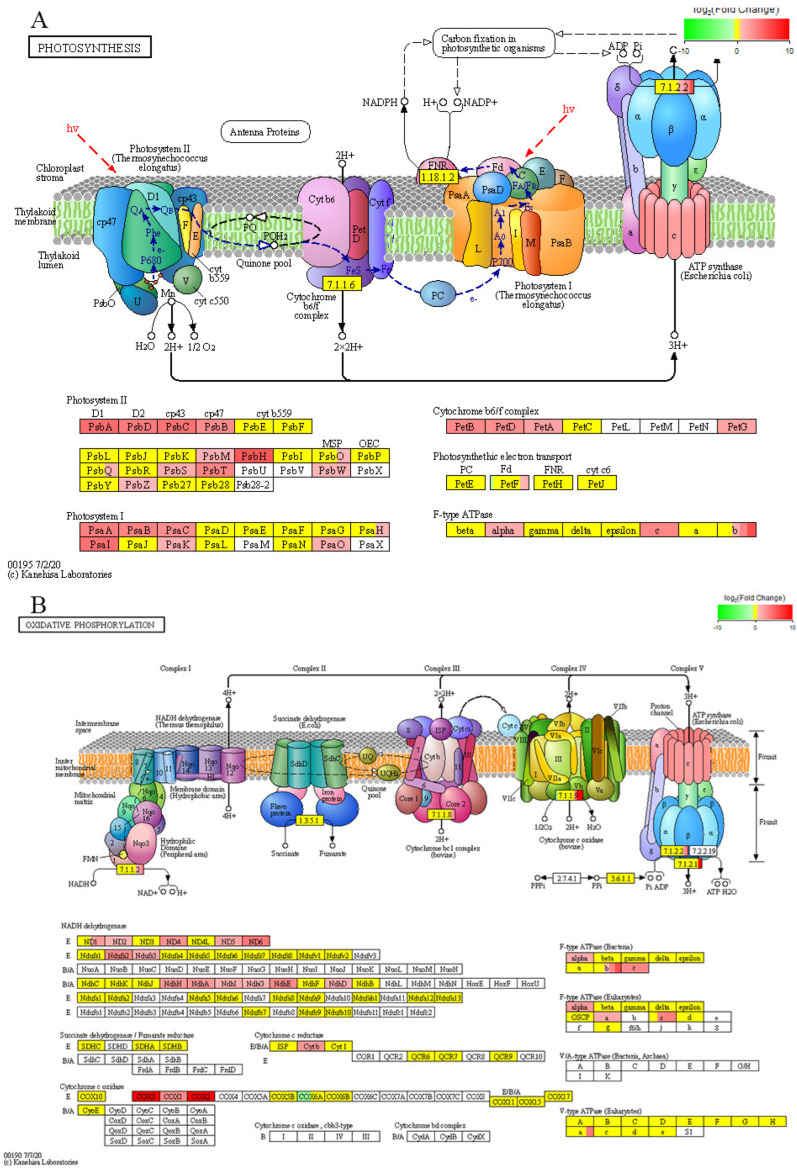

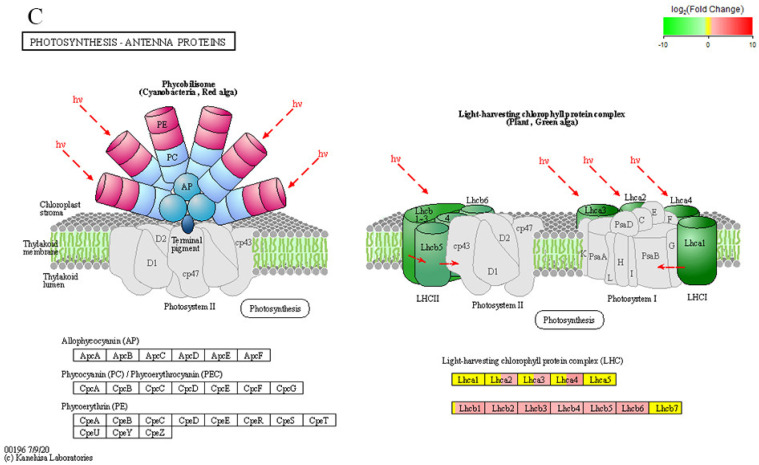
Enrichment maps for photosynthesis pathways in T-L vs. CK-L. (**A**) Photosynthesis pathway 1 (photosynthesis). (**B**) Photosynthesis pathway 2 (oxidative phosphorylation). (**C**) Photosynthesis pathway 3 (photosynthesis–antenna proteins).

Calcium ions act as secondary messengers involved in regulating photosynthesis during plant responses to environmental stress [23]. With in the photosynthesis pathway 1 (photosynthesis), the T-L vs. CK-L comparison showed 31 up-regulated DEGs (such as *PsbA* and *PsbB*) and 1 down-regulated DEG (*PetF*) (Figure 14A). With in the photosynthesis pathway 2 (oxidative phosphorylation), 29 up-regulated DEGs (such as *ND1* and *ND2)* and 1 down-regulated DEG (*COX6A*) (Figure 14B). In photosynthesis pathway 3 (photosynthesis–antenna proteins), 18 DEGs were up-regulated, including *LHca2* and *LHca3*, etc. (Figure 14C). These DEGs are responsive to BR under calcium deficiency stress in tomato leaves, causing significant changes in photosynthetic pathways under calcium deficiency stress, potentially affecting and regulating photosynthesis under such stress.

To validate the reliability of the RNA-Seq data, RT-qPCR analysis was performed on randomly selected up-regulated differentially expressed genes involved in metabolic pathways related to calcium uptake and distribution, the antioxidant system, and hormone signaling in the root, stem, and leaf tissues. The results showed that three out of the eight of the genes tested, Solyc06g048600.3, Solyc06g048930.3, and Solyc03g083870.3 were significantly up-regulated under BR treatment (Supplementary Figure S2). Although expression of the other five genes tested, Solyc01g110830.3, Solyc03g013460.1, Solyc03g116850.3, Solyc06g049010.2, and Solyc03g096670.3, was up-regulated, their relative transcript levels were increased considerably less than 2-fold.

## 3. Discussion

Tomato BER as a Classic Calcium-Related Physiological Disorder significantly interferes with tomato fruit yield and quality. Studying tomato plant responses to BER can provide valuable insights for developing strategies to reduce yield and quality losses caused by BER. The role of BR in regulating the growth and development of vegetable crops is significant. In field tomato production, the application of BR reduces the incidence of BER. This phenomenon may stem from the involvement of BR in tomato resistance to BER, suggesting that BR may play a key and positive role in triggering BER resistance in tomato plants. To validate this hypothesis, researchers conducted a study on the role of BR in the response to BER.

The maintenance of calcium homeostasis in plant cells relies on the coordinated action of various calcium transport proteins, including Ca^2+^/Cation antiporters (CaCAs), Ca^2+^ channels, and Ca^2+^-ATPases. Studies indicate that the Arabidopsis thaliana *AtNCL* protein, a type of Na^+^/Ca^2+^ exchanger localized on the vacuolar membrane, plays a crucial role in regulating intracellular calcium homeostasis. Its absence leads to abnormal plant growth and development and impaired stress responses [24,25]. Ho and White [14] pointed out that an absolute and critical fruit Ca^2+^ concentration threshold for BER occurrence has not been determined; BER can occur in fruits with seemingly adequate Ca^2+^ levels. Predicting and preventing BER in greenhouse tomatoes based solely on measurements of fruit Ca^2+^ status is not always effective. Some perspectives suggest that the issue might not be the total fruit Ca^2+^ content per se, but rather abnormal intracellular Ca^2+^ allocation and distribution leading to localized cellular Ca^2+^ deficiency, which subsequently triggers BER [14,26]. These research findings indicate that the relationship between calcium ions and tomato BER is not simply a matter of overall fruit calcium deficiency; the key lies in the imbalance of intracellular calcium ion homeostasis and abnormal spatial distribution.

In this study, it was found that under calcium-deficient conditions, foliar application of BR reduced the occurrence of BER. This aligns with the findings of Li Yutong et al. [21], where exogenous BR effectively reduced the incidence and severity index of tipburn in mini Chinese cabbage. Yan, Jingwei et al. [27] confirmed that BR treatment significantly increased the cytoplasmic Ca^2+^ concentration in maize mesophyll cell protoplasts, consistent with the findings of this study. BR treatment elevated calcium ion levels in leaves, stems, roots, and fruits, with significant increases observed in leaves, stems, and roots. Although the calcium content in fruits did not show a statistically significant rise, an increasing trend was noted. This might be due to calcium ion content being measured just as BER symptoms appeared, potentially explaining the lack of significant difference. This aligns with findings from Nonami H et al. [28], where Ca^2+^ concentrations were identical in all fruits across different tissue types before and immediately after BER symptoms appeared, with significant differences emerging only as BER further developed. This may imply that the arrangement structure of pectin in the middle lamella is crucial for regulating BER occurrence in plants. The results of this study suggest that BR may play a central role in triggering BER resistance responses. Notably, this research provides new insights into BR metabolism for controlling tomato BER occurrence, as there have been no prior reports mentioning that spraying BR reduces tomato BER.

BR can alleviate oxidative damage in plants under stress conditions by activating the antioxidant defense system. Key enzymes of this system, including CAT, POD, and SOD, collectively form an important line of defense for scavenging ROS in plants. Under drought stress, application of 0.1 µM BR significantly increased the activities of antioxidant enzymes, including CAT, POD, and SOD, in Oxalis corniculata, and significantly reduced H_2_O_2_ and MDA content [29]. Under moderate or severe water stress, 1-year-old Xanthoceras sorbifolia B. seedlings treated with 0.2 mg/L BR showed significantly higher activities of antioxidant enzymes (CAT, POD, and SOD) and lower MDA content compared to untreated seedlings [30]. Under salt stress, gourd treated with 0.208 µmol/L BR showed increased soluble sugar content, decreased free proline content, promoted activities of antioxidant enzymes (SOD, APX, and CAT), eliminated ROS damage, reduced MDA accumulation, maintained cell turgor pressure, and improved cell–environment osmotic adjustment [31]. Under drought conditions, foliar spraying with 20 mg/L BR significantly reduced the levels of H_2_O_2_ and MDA as well as electrolyte leakage in rassica juncea, while enhancing both the antioxidant system and photosynthetic efficiency [32]. These results indicate that appropriate concentrations of BR treatment under stress conditions can mitigate oxidative damage to plants. Our study results show that under calcium deficiency, H_2_O_2_ content accumulated significantly in leaves, while BR treatment reduced H_2_O_2_ content. MDA content also decreased with BR treatment, indicating that BR effectively mitigated oxidative damage to cell membranes.

BR has been demonstrated to enhance the expression of key genes involved in calcium signal transduction. For example, BR improved cold tolerance in tea plants by enhancing the expression of CaM, Calcium-Dependent Protein Kinase (CDPK), Calcineurin B-Like protein (CBL), and Calmodulin-Binding Transcription Activator (CAMTA) [33]. BR treatment improved chlorophyll fluorescence parameters, regulated the antioxidant system, alleviated oxidative damage induced by cold stress, and simultaneously promoted the up-regulation of ERF transcription factors and calcium-binding protein genes, enhancing grapevine tolerance to cold stress [34]. In our transcriptomic data, genes related to calcium-dependent proteins, calmodulin, and calcium-binding proteins were significantly up-regulated in leaves, stems, and roots. Particularly, the expression of the gene Solyc03g116850.3 was most significantly up-regulated in the roots, which might be a key molecular event in BR-regulated calcium signaling.

To further understand the physiological mechanism by which BR reduces BER occurrence, the gene expression patterns influenced by BR were investigated. Previous reports show that exogenous BR treatment in Mini Chinese cabbage up-regulated the relative expression levels of *BrACA4*, *BrACA11*, *BrECA1*, *BrECA3*, *BrECA4*, *BrCAX1*, *BrCAS*, and *BrCRT2* [21]. We hypothesized that spraying BR would reduce BER occurrence by altering the expression of disease resistance genes. This alteration in gene expression would enhance the plant’s ability to resist disease development, thus having a preventive effect before BER occurs. However, the effect of BR on gene expression in plants where BER was alleviated by BR application has not been studied [22]. In this study, BR up-regulated the expression of CaM, MYB, GST, COX, Cytochrome P450 members, and WRKY genes. CaM activates antioxidant enzymes like CAT3 to scavenge ROS [35]. Members of the MYB family enhance plant stress resistance by regulating the ABA signaling pathway and the synthesis of osmoregulatory substances (e.g., proline and betaine) [36]. Under stresses like drought and salinity, plants accumulate large amounts of ROS. Members of the GST family can directly utilize glutathione to reduce and scavenge hydrogen peroxide and lipid peroxides, etc., protecting cell membranes and proteins from oxidative damage [37]. COX transfers electrons to oxygen, generating water and releasing substantial energy used for ATP synthesis [38], thereby reducing intracellular free radical production and protecting cells from oxidative damage. Under 1 °C chilling injury conditions, BR significantly increased the activity of energy metabolism-related enzymes like COX in bamboo shoots [38]. In this study, the gene encoding COX, Solyc03g013460.1, showed high-magnitude up-regulation in roots, stems, and leaves, with an expression intensity gradient of roots > stems > leaves. Cytochrome P450 enzymes can synthesize flavonoids, lignin, and phytoalexins, etc., and directly scavenge ROS [39,40]. Members of the WRKY family participate in SA-mediated defense responses [41]. The CaMs, MYBs, GSTs, Cytochrome P450 members, and WRKYs whose expression increased in response to BR likely play positive roles in BR-induced BER resistance. Furthermore, these results provide new insights for regulating the expression of these genes.

Based on the above results, it can be concluded that BR plays a key role in tomato plant resistance to BER. The use of BR can significantly reduce BER incidence. BR enhances the comprehensive mechanism of tomato resistance to BER through a multi-level synergistic network of “promoting calcium absorption and transport-enhancing antioxidant defense-regulating energy and signaling pathways.” This provides an important theoretical basis and application strategy for using BR to prevent and control calcium-related physiological disorders in production.

## 4. Materials and Methods

### 4.1. Plant Material

This study used the flavor-type tomato variety ‘Nongbo Fen 18109’, bred by the Shijiazhuang Academy of Agriculture and Forestry Sciences.

### 4.2. Experimental Design

The experiment was conducted in 2024 in a solar greenhouse at the Zhao County Experimental Base of the Shijiazhuang Academy of Agriculture and Forestry Sciences, located at 114°49′26″ E, 37°49′59″ N. The site experiences a warm temperate semi-humid continental monsoon climate with an annual average temperature of 12.5 °C. A trough-based vermiculite cultivation system was used, with seedlings raised uniformly. Two treatments were established: calcium-deficient nutrient solution cultivation + distilled water spray (CK, control) and calcium-deficient nutrient solution cultivation + BR spray (T). Each treatment had three replicates. The nutrient solution was based on the Hoagland formula with calcium omitted, maintaining a pH of 6.0~6.5, an EC of 2.0~2.5 ms/cm, a temperature range of 12~35 °C, and a photoperiod of 10~12 h. On 6 August, seedlings at the three-true-leaf stage were transplanted into cultivation troughs with a plant spacing of 40 cm. Fertilization began after the seedlings had acclimatized. Foliar spraying of BR commenced on 29 August, after the first flower cluster had bloomed. The BR concentration was 0.1 mg/L, determined as the optimal concentration for controlling tomato BER in prior screening experiments. Spraying was performed every three days for a total of 10 applications, with leaves sprayed until droplets formed on all leaf surfaces. Samples were collected 50 days after the first BR application. Leaves, stems, and roots were sampled from each of the three replicates. Immediately after collection, samples were flash-frozen in liquid nitrogen and stored at −80 °C for later analysis. The entire experiment was repeated three times.

### 4.3. Determination of Antioxidant Enzyme Activities

The activities of catalase (CAT), peroxidase (POD), superoxide dismutase (SOD), and the contents of malondialdehyde (MDA) and hydrogen peroxide (H_2_O_2_) in tomato leaves were determined using kits (Solarbio, Beijing, China) by Nanjing ProNet Biotech Co., Ltd. (Nanjing, China).

Approximately 0.1 g of tomato leaf tissue was weighed and homogenized in an ice bath with 1 mL of phosphate-buffered saline (PBS, 100 mmol/L, pH 7.8). After centrifugation at 4 °C and 8000 rpm for 10 min, the supernatant was collected as the test sample solution.

CAT assay: 20 μL of sample was mixed with 100 μL of 100 mM Tris buffer (pH 7.8), incubated in a 25 °C water bath for 10 min; then, 100 μL of (NH_4_)_2_MoO_4_ solution was added. After reacting for 10 min, the absorbance at 405 nm was measured using a microplate reader for the enzyme activity calculation.

POD assay: A working solution was prepared by mixing the kit’s PBS, 2-methoxyphenol, and 30% H_2_O_2_ in a ratio of 2.6 (mL):1.5 (μL):1 (μL), followed by incubation at 25 °C for 10 min. In a 96-well plate, 10 μL of sample solution and 190 μL of working solution were mixed and timed. The absorbance at 470 nm was recorded at 1 min (A_1_) and 2 min (A_2_) using a microplate reader for the enzyme activity calculation.

SOD assay: 50 μL of supernatant was mixed with 1.5 mL of 130 mmol/L methionine (Met) solution, 0.3 mL of 100 μmol/L EDTA-Na_2_, 0.3 mL of 20 μM riboflavin solution, and 0.3 mL of 750 μmol/L nitroblue tetrazolium (NBT). After thorough mixing and standing at room temperature for 30 min, the absorbance at 506 nm was measured for the enzyme activity calculation.

MDA assay: 1 mL of enzyme extract was mixed with 2 mL of 0.6% TBA, sealed, and heated in a boiling water bath for 15 min. After rapid cooling and centrifugation, the supernatant was collected. Absorbance was measured at 600, 532, and 450 nm to calculate MDA content.

H_2_O_2_ assay: The supernatant was treated with titanium sulfate and concentrated ammonia, centrifuged for 10 min. The precipitate was washed with acetone by shaking and centrifugation, then dissolved in 2 mol·L^−1^ H_2_SO_4_. The OD value was measured at 415 nm.

### 4.4. Tomato Sample Total RNA Extraction, Library Construction, and Transcriptome Sequencing

Transcriptome sequencing was performed by Sangon Biotech (Shanghai) Co., Ltd. (Shanghai, China). After RNA extraction, purification, and library construction, Illumina sequencing was conducted. Raw sequencing data quality was assessed using FastQC 0.11.2, and quality trimming was performed using Trimmomatic 0.36 to obtain clean data. The HISAT2 2.1.0 software was used to align the quality-controlled sequencing reads to the tomato reference genome (https://www.ncbi.nlm.nih.gov/datasets/genome/?taxon=4081 accessed on 25 November 2024) for differential gene expression analysis to obtain DEGs. DEGs were filtered and subjected to GO and KEGG pathway enrichment analyses, referring to the methods of Van der Auwera, G.A. et al. and Damian, S. et al. [42,43].

### 4.5. Combined Transcriptomic and qRT-PCR Approach Identifies and Validates Tissue-Specific Core Genes

Differential expression analysis between the treatment group (T) and control group (CK) for leaves (T-L vs. CK-L), stems (T-S vs. CK-S), and roots (T-R vs. CK-R) was performed using DESeq2. GO enrichment and KEGG pathway enrichment analyses were conducted. Core DEGs most related to BR regulation under calcium deficiency stress and metabolic pathways in different tomato parts were identified. A total of ten core DEGs were identified from roots, stems, and leaves and validated by qRT-PCR, with nine technical replicates and three biological replicates performed for each gene. The results are presented in Supplementary Figure S2. These core DEGs were validated by qRT-PCR, referring to the method of Ashburner et al. [44]. Primer design followed the method of Shannon P [45]. The primer list is provided in Supplementary Table S1.

### 4.6. Data Analysis

R language4.5 was used for differential data analysis, and Origin 2024 was used for plotting the differential analysis results.

## Figures and Tables

**Table 1:** DEGs in T-L vs. CK-L.

Up-Regulated Gene Annotation Group	Number of Genes
Calmodulin (CAM)	1
MADS-box transcription factor	1
MYB transcription factors	3
Cold shock protein	1
Glycine-rich protein	6
Glutathione S-transferase (GST)	4
Pectinesterase	4
COX	4
Fatty acyl-coa	2
ABC transporter	3
ABA deficient protein	1
Sugar transport proteins	3
Auxin-responsive protein SAUR	5
Ribosomal proteins	6
Auxin efflux facilitator slpin	1
Atpase	8
Cytochrome P450	5
Other	414
Down-regulated gene annotation group	
Heat shock protein	3
MADS-box protein	1
MYB transcription factors	2
Glycine-rich protein	2
Cytochrome P450	13
Jasmonic acid-amido	1
Glucan endonuclease	6
GST	1
Pathogenesis-related leaf protein	4
Sugar transport proteins	2
Ethylene-responsive transcription factors	8
WRKY transcription factors	6
Serine/threonine protein kinases	8
Della protein	1
Other	459

**Table 2:** DEGs in T-S vs. CK-S.

Up-Regulated Gene Annotation Group	Number of Genes
GST	1
Cytochrome P450	2
COX	1
Pectinesterase	1
Calcium-Binding Protein	1
Sugar Transport Proteins	3
Ribosomal Protein	1
Auxin	3
Stress-Associated Protein	3
Ethylene-Responsive Transcription Factors	3
Other	222
Down-regulated gene annotation group	
Calcium-binding protein	3
MYB transcription factors	9
WRKY transcription factors	8
Cytochrome P450	7
Auxin	25
GST	2
Pectinesterase	1
Serine/threonine protein kinases	7
Ethylene-responsive transcription factors	19
Jasmonic acid-amido	1
Sugar transport proteins	2
B3 domain-containing proteins	2
Bhlh transcription factors	3
Other	344

**Table 3:** DEGs in T-R vs. CK-R.

Up-Regulated Gene Annotation Group	Number of Genes
WRKY transcription factors	2
Calcium-binding protein	1
MYB transcription factors	5
ABC transporter	1
Ribosomal protein	1
Sugar transport proteins	8
Bhlh transcription factors	4
Glycine-rich proteins	2
Pectinesterase	4
Della proteins	2
Other	436
Down-regulated gene annotation group	
Sugar transport proteins	2
MYB transcription factor	1
Auxin-responsive proteins	3
ABC transporter	3
Bhlh transcription factor	1
Pectinesterase	4
Ethylene-responsive transcription factor	1
Other	279

**Table 4:** GO enrichment of DEGs in different comparison groups.

Category	DEGs	GO Enrichment			
		Biological Process	Cellular Component	Molecular Function	Total
T-L vs. CK-L	4807	1654	1574	1422	4650
T-S vs. CK-S	2807	986	914	864	2764
T-R vs.CK-R	2554	853	846	759	2458

## Data Availability

Data available on request from the authors. The data are not publicly available due to privacy or ethical restrictions.

## Supplementary Materials

The following supporting information can be downloaded at: https://www.mdpi.com/article/10.3390/plants15030427/s1, Figure S1: Transcriptome data PCA plot: PC1 accounts for 56.4%, PC2 for 23.52%, and PC3 for 12.75%; Figure S2: Ten core DEGs validated by qRT-PCR (ΔΔCt method); Table S1: Sequence of the primers used in this work.
