# Supplementary material for: Brassinolide Application Mitigates Blossom-End Rot in Tomato by Enhancing Calcium Homeostasis and Antioxidant Defense Under Calcium Deficiency
Source: Plants (Basel). 2026 Jan 30;15(3):427. doi: 10.3390/plants15030427 (PMC12899996; doi:10.3390/plants15030427)
Supplement: Supplementary file 1 [file plants-15-00427-s001.zip › Supplementary-Figures.pdf]

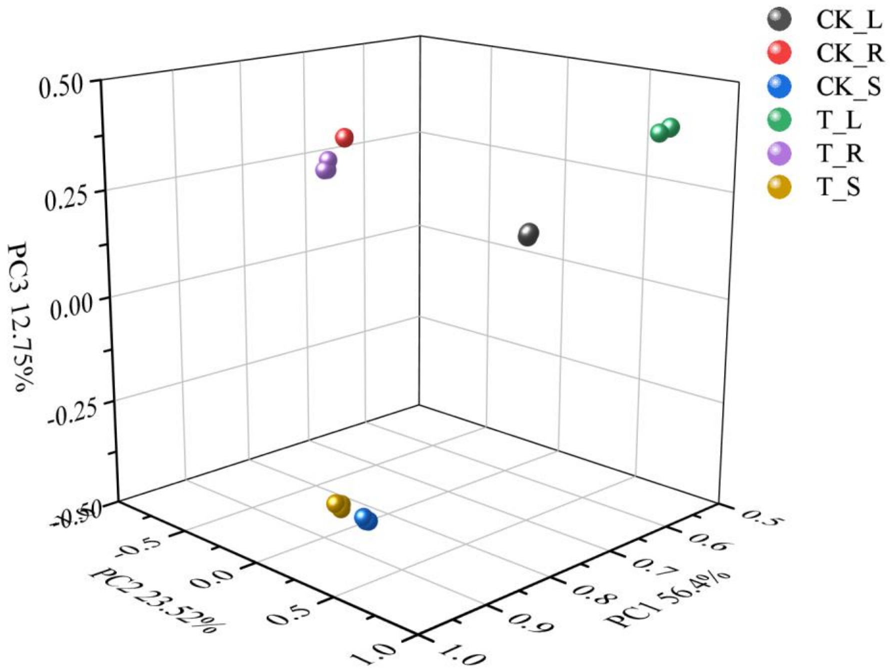

Figure S1 Transcriptome data PCA plot: PC1 accounts for 56.4%, PC2 for 23.52%, and PC3 for 12.75%.

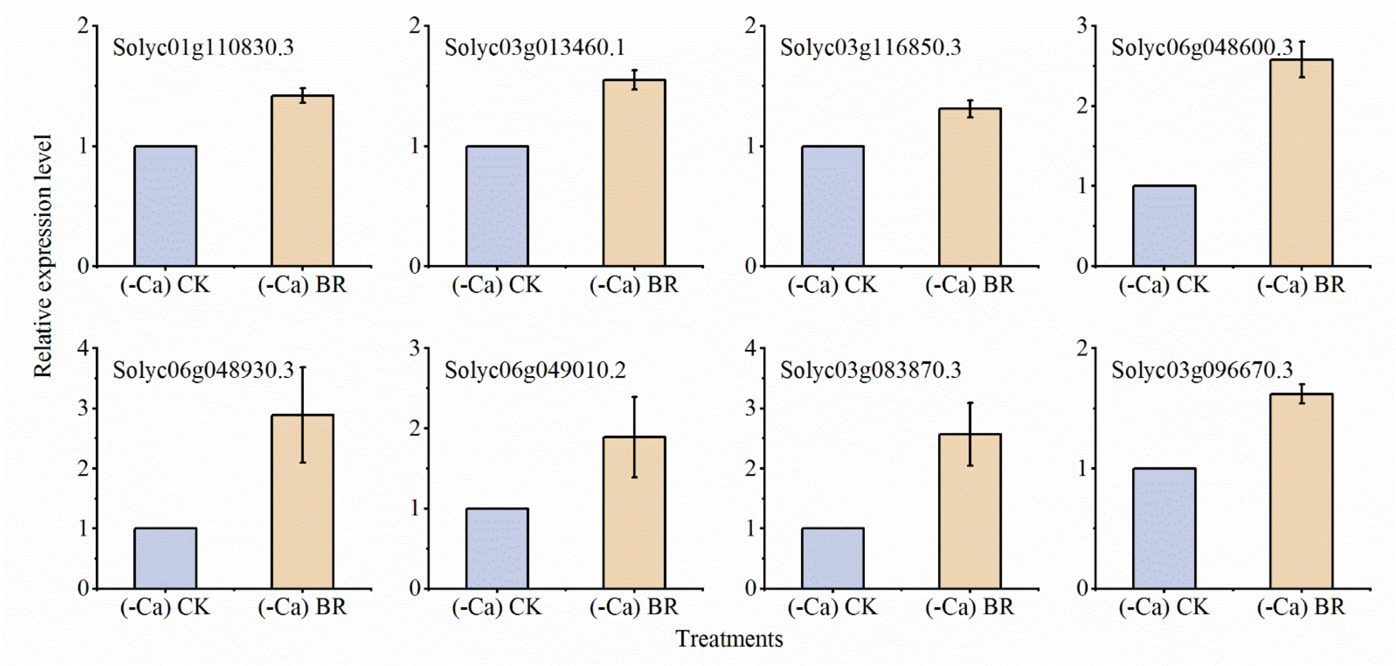

Figure S2 Ten core DEGs validated by qRT-PCR (ΔΔCt method).
